# Enzyme–DNA Interactions Affect the Catalytic Inhibition of Mycobacterial Gyrases by Antibacterial Drugs

**DOI:** 10.1021/acsinfecdis.5c01018

**Published:** 2026-01-27

**Authors:** Jillian F. Armenia, Neil Osheroff

**Affiliations:** † Department of Biochemistry, 12327 Vanderbilt University School of Medicine, Nashville, Tennessee 37232, United States; ‡ Department of Medicine (Hematology/Oncology), Vanderbilt University School of Medicine, Nashville, Tennessee 37232 United States

**Keywords:** gyrase, DNA decatenation, DNA supercoiling, DNA relaxation, fluoroquinolones, spiropyrimidinetriones

## Abstract

Gyrase and topoisomerase IV, enzymes that play critical roles during DNA replication, are the targets of fluoroquinolones and other antibacterials. Gyrase removes positive supercoils that accumulate ahead of replication forks, while topoisomerase IV untangles daughter chromosomes. Although topoisomerase IV is an essential enzyme in most bacteria, some species, including *Mycobacterium tuberculosis* and *Mycobacteroides abscessus*, encode only gyrase. In these species, gyrase is the sole target of fluoroquinolones and is believed to assume the cellular functions of both type II topoisomerases. Although fluoroquinolones and emerging antibacterials such as spiropyrimidinetriones induce gyrase-mediated DNA cleavage, there is evidence that inhibition of gyrase function also plays a role in drug-induced cell death under some circumstances. Therefore, we examined the effects of moxifloxacin and ciprofloxacin (fluoroquinolones) and zoliflodacin (spiropyrimidinetrione) on the three catalytic activities presumably carried out by gyrase in mycobacteria: decatenation of tangled DNA, negative supercoiling of relaxed DNA, and relaxation of positive supercoils. Under all circumstances, lower concentrations of antibacterials were required to inhibit intermolecular DNA decatenation as compared to the intramolecular DNA relaxation or supercoiling functions of gyrase. Differences in drug potency could not be attributed solely to rates of individual reactions or the DNA substrates utilized. Rather, results suggest that the potency of gyrase inhibition by interfacial antibacterials is modulated by the topological state of the DNA and its specific interactions with gyrase. Whereas most studies focus on DNA cleavage induced by gyrase-targeted antibacterials, this study provides mechanistic insights into how antibacterials rob replicating cells of essential gyrase functions.

## Graphical abstract

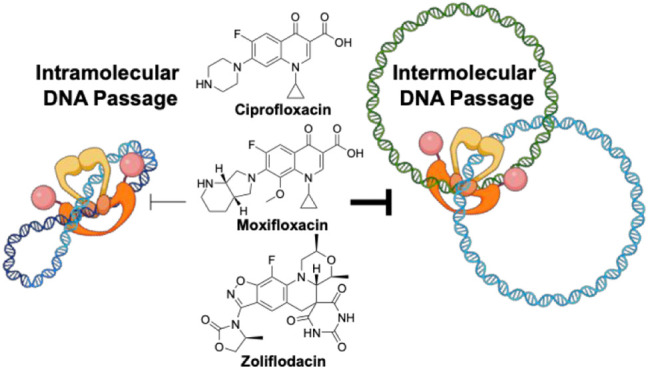

Type II topoisomerases are essential enzymes across all domains of life.^1–4^ These enzymes play critical roles in regulating levels of DNA under- and overwinding and remove knots and tangles from the genome. Typically, bacteria encode two type II topoisomerases, gyrase and topoisomerase IV.^5–7^ Gyrase is the only known enzyme that can independently introduce negative supercoils into (i.e., underwind) relaxed DNA.^8^ It also removes positive supercoils (overwound DNA) that are generated in front of DNA tracking systems such as replication and transcription complexes.^9,10^ Topoisomerase IV can remove positive supercoils (albeit not as rapidly as gyrase) or negative supercoils from DNA.^11–13^ However, its primary cellular task is to untangle (i.e., decatenate) daughter chromosomes following replication and remove DNA knots that occur as a result of DNA recombination/repair pathways.^14–17^

Gyrase and topoisomerase IV share a common catalytic mechanism. Both enzymes alter the topology of DNA by passing an intact double helix (the transport, or T-segment) through a transient double-stranded break that they generate in a different section of DNA (the gate, or G-segment).^1–3,7,18–20^ To maintain genomic integrity during the double-stranded DNA passage reaction, the enzymes form covalent bonds between active site tyrosine residues and the 5′ phosphate termini of the cleaved DNA.

What distinguishes gyrase and topoisomerase IV, and leads to the differences in their primary cellular functions, is the ability of gyrase to wrap DNA around its C-terminal domain.^6,21,22^ This wrapping mechanism is facilitated by the GyrA-box, a seven-amino acid motif in the C-terminal domain that is essential for the DNA supercoiling reaction catalyzed by gyrase.^23^ DNA wrapping results in the formation of a continuum between the G- and T-segments and creates a positive supercoil in the DNA which is converted to a negative supercoil following the strand passage event. DNA wrapping gives gyrase a unidirectional mechanism, which allows the enzyme to rapidly remove positive supercoils and introduce negative supercoils into DNA.^13,24^ In addition, DNA wrapping by the C-terminal domain causes gyrase to strongly favor catalysis of intramolecular strand passage reactions (relaxation and supercoiling) over intermolecular strand passage reactions (catenation and decatenation).^21^

Unlike gyrase, the C-terminal domain of topoisomerase IV does not contain the conserved GyrA-box motif and is incapable of wrapping DNA.^6,25^ Consequently, the enzyme can remove both positive and negative DNA supercoils.^13^ More importantly, this lack of wrapping facilitates the ability of topoisomerase IV to carry out strand passage reactions between two separate DNA molecules.^14–16^ This enables the enzyme to decatenate daughter chromosomes following DNA replication. Topoisomerase IV is an essential enzyme in bacterial species that encode both type II topoisomerases, and mutations that undercut its catalytic activity lead to severe chromosome partitioning defects.^14^

Given the importance of topoisomerase IV for cell viability, it is surprising that a number of bacterial species encode only one type II topoisomerase, gyrase. Among these bacteria are *Mycobacterium tuberculosis* (the causative agent of tuberculosis) and the nontuberculous mycobacterium *Mycobacteroides abscessus* (which primarily causes lung infections in immunocompromised individuals).^26,27^ Note that *M. abscessus* is often referred to by its legacy name: *Mycobacterium abscessus*. Based on their genome sequences, these species encode no other known topoisomerases capable of decatenating daughter chromosomes.^26,27^ Consequently, in these species, and others that encode only gyrase, it is believed that the enzyme functions as a hybrid type II topoisomerase that carries out both the intramolecular strand passage roles of gyrase and the intermolecular strand passage roles of topoisomerase IV.^26,28–30^

Beyond the catalytic functions of gyrase and topoisomerase IV, these enzymes are also the targets for fluoroquinolone antibacterials and emerging classes of antibacterial compounds such as spiropyrimidinetriones (SPTs).^20,31–35^ These interfacial antibacterials (i.e., antibacterials that interact at the interface of the enzyme and DNA)^36^ insert themselves into the cleaved scissile bonds following cleavage of the G-segment.^20,32,34–37^ This results in two potentially deleterious effects, both of which are capable of killing bacterial cells. First, stabilization of the covalent enzyme-cleaved DNA complex can lead to fragmentation of the genome. Second, this stabilization inhibits the overall catalytic activity of the type II topoisomerases, robbing the cell of essential enzymatic functions. There is evidence for both mechanisms of antibacterial activity contributing to cell death.^37,38^

In species that encode both type II topoisomerases, impairment of gyrase activity hinders cell growth by slowing DNA replication,^7,39–41^ whereas inhibition of topoisomerase IV impedes chromosome segregation, which leads to catastrophic cell division.^7,14,16,41^ The effects of antibacterials on the enzymatic activity of purified gyrase are virtually always assessed by determining the effects of compounds on rates of DNA negative supercoiling. This ignores the temporally more important function of gyrase, the removal of positive supercoils.^13,42^ For topoisomerase IV, antibacterial effects on catalytic activity are generally assessed by the inhibition of decatenation.^43,44^ However, the inhibition of these different catalytic activities has not been assessed in species such as *Mycobacteria*, in which gyrase is presumably catalyzing all three reactions.

The most extensively studied gyrase from a species that does not encode topoisomerase IV is the enzyme from *M. tuberculosis*.^26,29,42,45,46^ It has also been demonstrated in this species that under conditions of low gyrase activity, cells become hypersensitive to fluoroquinolones and SPTs.^37^ This finding suggests that, under at least some circumstances, the inhibition of gyrase activity plays a more important role in antibacterial-induced cell death than the conversion of gyrase to an enzyme that fragments the genome.

The goal of this work was to determine which reactions carried out by mycobacterial gyrase were most susceptible to the inhibitory effects of antibacterials. Therefore, we assessed the effects of fluoroquinolones and SPTs on three catalytic activities of mycobacterial gyrases *in vitro*: relaxation of positive supercoils, introduction of negative supercoils into relaxed DNA, and decatenation of kinetoplast DNA (kDNA). Throughout the study, gyrase from *M. tuberculosis* was examined and gyrase from *M. abscessus* (a nontuberculous mycobacterium) was used as a comparator to make the results more generalizable.

Ultimately, fluoroquinolones and SPTs inhibited intermolecular DNA decatenation at lower concentrations than were required to inhibit either of the intramolecular functions of gyrase (relaxation of positive supercoils or introduction of negative supercoils). Results with wild-type gyrases and enzymes with mutated GyrA-boxes that were incapable of wrapping DNA indicate that while reaction rate may influence the differential inhibition of various gyrase catalyzed reactions, additional factors related to DNA topology appear to also play an important role. Finally, our results predict that inhibition of gyrase by interfacial antibacterials in species that do not encode topoisomerase IV may lead primarily to defects in chromosome partitioning as opposed to decreased rates of DNA replication.

## Results

### Rates of Gyrase-Catalyzed Strand Passage Reactions

As a prelude to catalytic inhibition experiments with antibacterial drugs, we established a single set of reaction conditions, except for the DNA substrate, under which we could measure all the catalytic functions of gyrase. We used these conditions to compare the rates of relaxation of positive supercoils, introduction of negative supercoils into relaxed DNA, and decatenation of kinetoplast DNA by *M. tuberculosis* and *M. abscessus* gyrase (Figure 1). We utilized the loss or gain of the fully supercoiled band, respectively, to monitor rates of relaxation of positive supercoils or introduction of negative supercoils into relaxed DNA. It is notable that neither reaction is completely processive (i.e., intermediate bands are observed), and that monitoring the fully supercoiled band alone does not provide a rate that is fully reflective of the activity of the enzyme. However, these rates provide a relative basis for direct comparisons between reactions.

**Figure 1:**
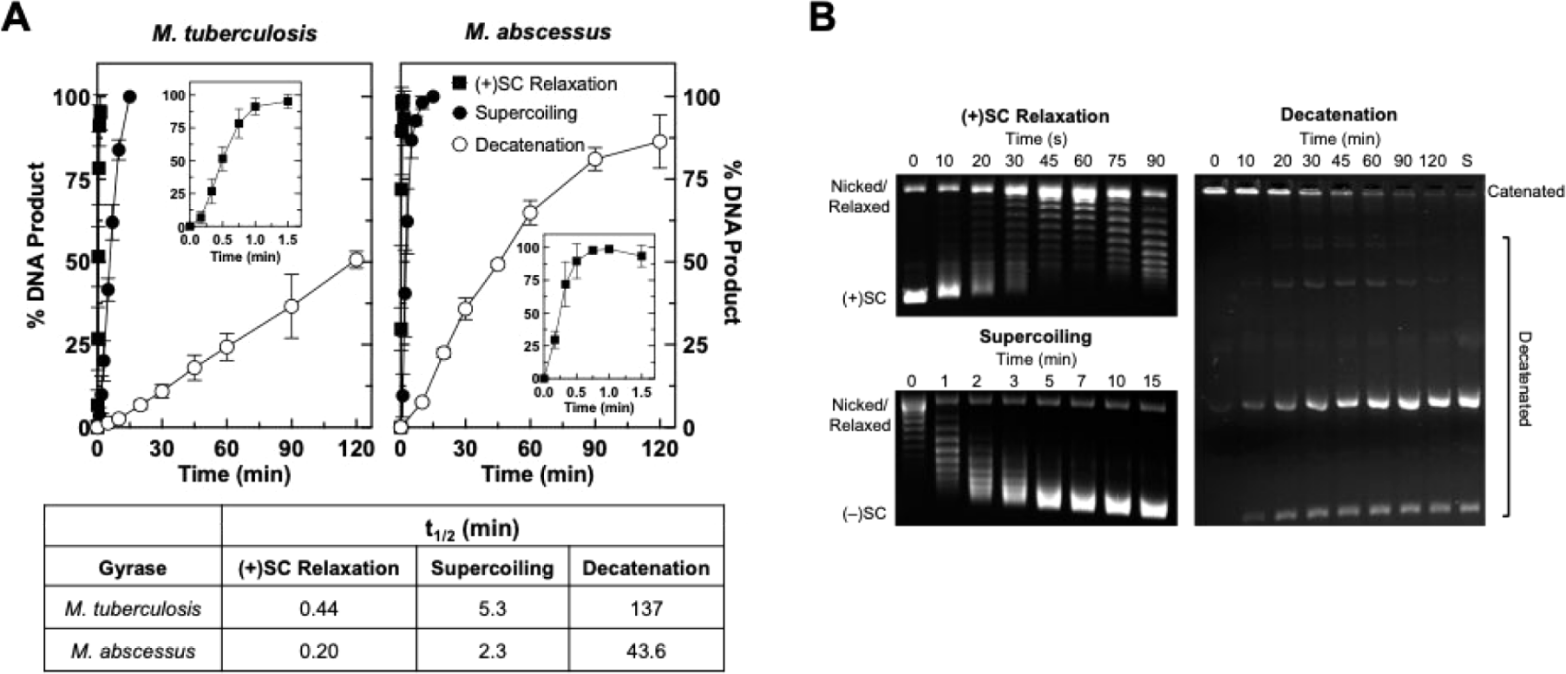
*In vitro* activities of gyrase from *M. tuberculosis* and *M. abscessus*. (A) Time courses for the rates of removal of positive supercoils [ (+)­SC Relaxation, closed squares], introduction of negative supercoils (closed circles, Supercoiling), and decatenation of kDNA (Decatenation, open circles) by *M. tuberculosis* (left) and *M. abscessu* *s* gyrase (right). Insets show (+)­SC relaxation experiments on a shorter time scale. Error bars represent the standard deviation of at least three independent experiments. The table at the bottom of panel A shows the half-times (*t*_1/2_) for each reaction in minutes. (B) Representative gel images for each catalytic function. (+)­SC relaxation (top left) was quantified as the loss of the substrate present at time zero. Supercoiling (bottom left) was quantified as a percentage of the fully supercoiled DNA at the final time point. Decatenation (right) was quantified as a percentage based on a standard lane (S) containing 0.3 μg of fully decatenated kDNA.

In agreement with previous studies,^13,42^ gyrase from either species removed positive supercoils considerably faster than it introduced negative supercoils into relaxed DNA. Furthermore, decatenation catalyzed by gyrase was considerably slower than either of the intramolecular strand passage reactions. With reaction conditions established, we compared the effects of gyrase-targeted antibacterials on the different catalytic functions of gyrase.

### Catalytic Inhibition of Purified Wild-Type *M. tuberculosis* and *M. abscessus* Gyrase by Fluoroquinolones

Fluoroquinolones are among the world’s most efficacious and broad-spectrum oral antibacterials and have been in clinical use since the 1980s.^31,34,47–49^ Two fluoroquinolone molecules stabilize the gyrase-cleaved DNA complex by forming a water-metal ion bridge with two highly conserved amino acid residues in the A subunit of gyrase and interacting with DNA by inserting between the DNA bases at the cleaved scissile bonds.^31,34,35,50–54^

Fluoroquinolones, including moxifloxacin and ciprofloxacin, have been employed as part of treatment regimens for mycobacterial infections. Moxifloxacin was recently recommended by the World Health Organization and the Centers for Disease Control and Prevention as part of new regimens for the treatment of tuberculosis.^55,56^ Therefore, the effects of moxifloxacin and ciprofloxacin on the three catalytic activities of purified *M. tuberculosis* and *M. abscessus* gyrase were determined.

With both enzymes, moxifloxacin (Figure 2) inhibited decatenation at the lowest concentration, followed, in order, by supercoiling of relaxed DNA and relaxation of positive supercoils. This result affords a potentially straightforward explanation in which inhibition correlates with the rates of the individual reactions. In this explanation, decatenation is inhibited at the lowest concentration of moxifloxacin because fewer rounds of catalysis can take place during the time that the drug molecules are unbound. Conversely, in a faster reaction, more rounds of catalysis take place in the time that gyrase is drug free. Thus, relaxation of positive supercoils requires the most drug to inhibit.

**Figure 2:**
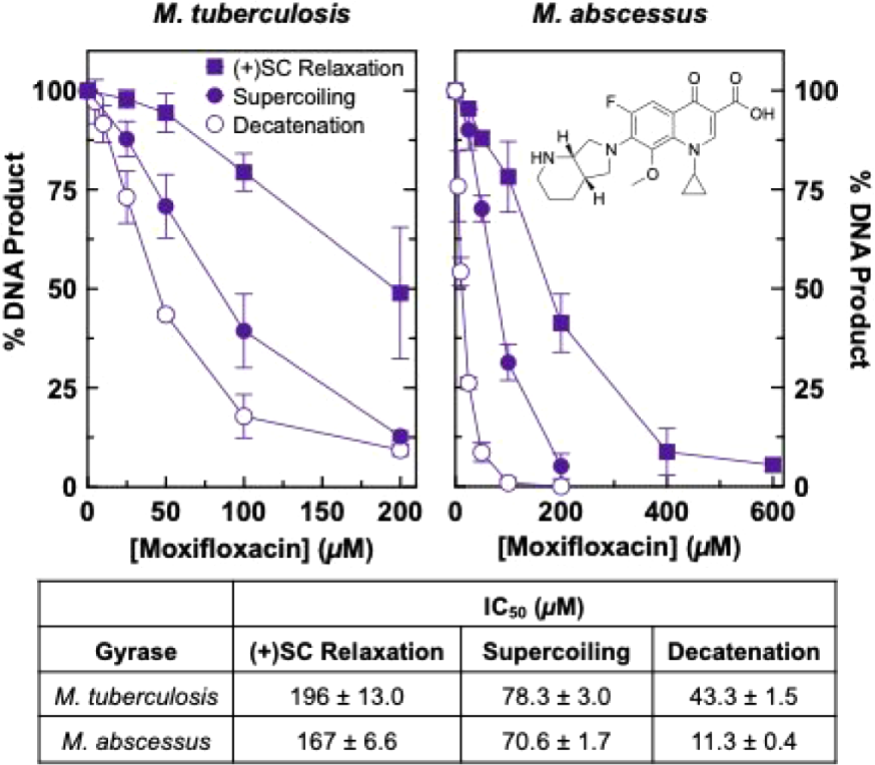
Inhibition of the three catalytic activities of *M. tuberculosis* and *M. abscessus* gyrase by moxifloxacin. The ability of *M. tuberculosis* (left panel) and *M. abscessus* (right panel) gyrase to catalyze decatenation of kDNA (Decatenation, open circles), negative supercoiling of relaxed DNA (Supercoiling, closed circles), and relaxation of positive supercoils [(+)­SC Relaxation, closed squares] in the presence of increasing concentrations of moxifloxacin is shown. The structure of moxifloxacin is shown in the right panel. Error bars represent the standard deviations of at least three independent experiments. IC_50_ values (the concentration of drug required to inhibit 50% of gyrase activity) are listed in μM with the standard error of the mean in the table at the bottom of the figure.

In contrast, this potentially straightforward explanation does not hold for the inhibition of *M. tuberculosis* and *M. abscessus* gyrase activities by ciprofloxacin (Figure 3). Whereas inhibition of intermolecular decatenation still required the lowest concentration of drug, inhibition of the two intramolecular reactions (supercoiling of relaxed DNA and relaxation of positively supercoiled DNA) was achieved at similar concentrations of ciprofloxacin. This is despite the fact that the rates of DNA supercoiling and relaxation differ by more than an order of magnitude (see Figure 1). These findings suggest that a simple correlation between reaction rate and drug potency is not sufficient to explain the observed differences in catalytic inhibition by fluoroquinolones.

**Figure 3:**
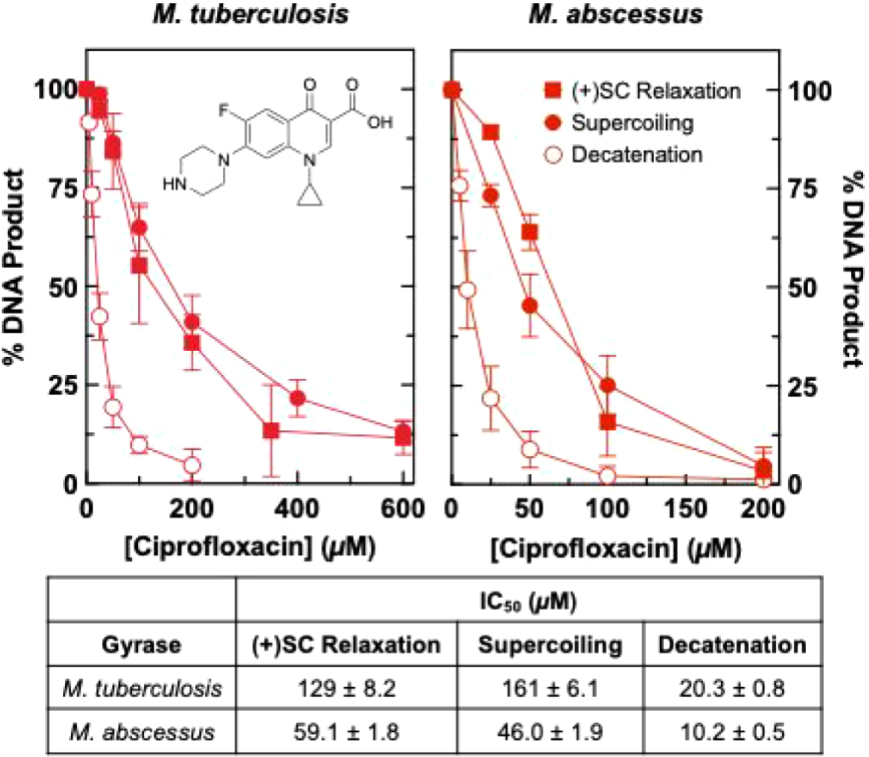
Inhibition of the three catalytic activities of *M. tuberculosis* and *M. abscessus* gyrase by ciprofloxacin. The ability of *M. tuberculosis* (left panel) and *M. abscessus* (right panel) gyrase to catalyze decatenation of kDNA (Decatenation, open circles), negative supercoiling of relaxed DNA (Supercoiling, closed circles), and relaxation of positive supercoils [(+)­SC Relaxation, closed squares] in the presence of increasing concentrations of ciprofloxacin is shown. The structure of ciprofloxacin is shown in the left panel. Error bars represent the standard deviation of at least three independent experiments. IC_50_ values are listed in μM with the standard error of the mean in the table at the bottom of the figure.

### Catalytic Inhibition of Purified Wild-Type *M. tuberculosis* and *M. abscessus* Gyrase by Zoliflodacin

Zoliflodacin is a first-in-class spiropyrimidinetrione (SPT) that was recently approved by the United States Food and Drug Administration for the treatment of uncomplicated urogenital gonorrhea.^57,58^ Like fluoroquinolones, zoliflodacin interacts with both gyrase and DNA to stabilize the covalent enzyme-cleaved DNA complex and acts by inserting two drug molecules, one at each cleaved scissile bond.^59^ Although the SPT interacts with different amino acid residues than fluoroquinolones, the two drug classes share overlapping binding pockets on gyrase and are mechanistically similar.^34,35,59^

Because moxifloxacin and ciprofloxacin displayed different patterns of inhibition, we determined the effects of zoliflodacin on the three catalytic functions of gyrase. As seen in Figure 4, the inhibition pattern of zoliflodacin was identical to that of ciprofloxacin. Decatenation was inhibited at the lowest concentration of zoliflodacin, whereas relaxation of positively supercoiled DNA and introduction of negative supercoils into relaxed DNA were inhibited at similar concentrations. Once again, this finding suggests that reaction rates are not the sole determinant of catalytic inhibition.

**Figure 4:**
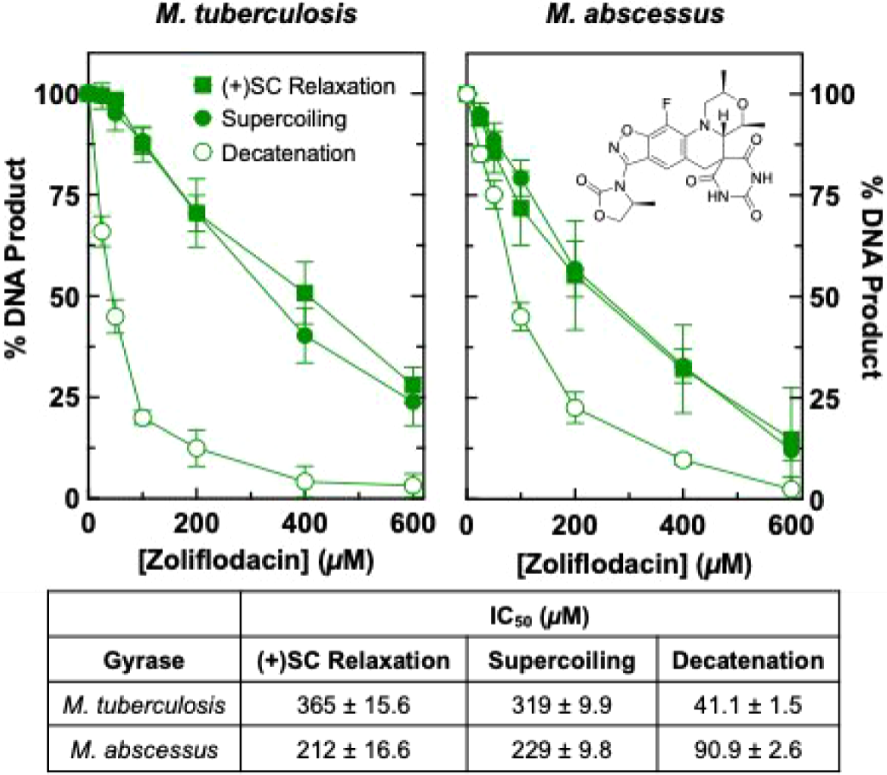
Inhibition of the three catalytic activities of *M. tuberculosis* and *M. abscessus* gyrase by zoliflodacin. The ability of *M. tuberculosis* (left panel) and *M. abscessus* (right panel) gyrase to catalyze decatenation of kDNA (Decatenation, open circles), negative supercoiling of relaxed DNA (Supercoiling, closed circles), and relaxation of positive supercoils [(+)­SC Relaxation, closed squares] in the presence of increasing concentrations of zoliflodacin is shown. The structure of zoliflodacin is shown in the right panel. Error bars represent the standard deviation of at least three independent experiments. IC_50_ values are listed in μM with the standard error of the mean in the table at the bottom of the figure.

### Effects of DNA Wrapping on the Catalytic Inhibition of Mycobacterial Gyrase

The above data indicate that drug potency is based on more than reaction rates alone and implies that drugs may affect inter- (decatenation) and intramolecular (supercoiling and relaxation) DNA passage reactions differently. However, there is a caveat to this explanation. The decatenation assay uses kDNA, which is a network of interlinked DNA maxi- and minicircles that is isolated from the kinetoplasts of *Crithidia fasciculata*.^60^ This is in contrast to the DNA relaxation and supercoiling reactions, which use a monomeric plasmid (pBR322) isolated from *Escherichia coli*. Furthermore, it is not known how many rounds of catalysis are required to release individual circles from the kDNA network. Therefore, to eliminate this potentially confounding variable, we sought to establish a decatenation system in which reaction rates could be varied while using the same DNA substrate. To this point, we speculated that a mutation in gyrase that eliminated the ability of the enzyme to wrap DNA might enhance its ability to carry out intermolecular reactions and thereby increase the rate of decatenation.

A previous study with *Escherichia coli* gyrase demonstrated that deletion of the C-terminal domain of the GyrA subunit (the portion of the enzyme responsible for wrapping DNA) abolished the ability of gyrase to introduce negative supercoils into relaxed DNA and enhanced the ability of the enzyme to decatenate DNA.^21^ A later study identified a conserved 7-amino acid motif, the GyrA-box [Q­(R/K)­RGG­(R/K)­G], in the C-terminal domain of GyrA that is essential for DNA wrapping and demonstrated that the conversion of this motif to alanine residues was sufficient to abrogate DNA wrapping and supercoiling activity of *E. coli* gyrase.^23^ The importance of the GyrA-box for the interaction of gyrase with DNA has also been confirmed by structural and single-molecule studies.^61,62^ Finally, a study with *M. tuberculosis* gyrase showed that substitution of the three glycine residues with alanine residues was sufficient to eliminate the DNA supercoiling activity of the enzyme.^46^

With the above in mind, we recapitulated the three glycine to alanine substitutions (GyrA-3xA) in the GyrA-box of *M. tuberculosis* gyrase and made the identical substitutions in the GyrA-box of *M. abscessus* gyrase. The activities of these enzymes were analyzed by two-dimensional gel electrophoresis to resolve positively vs negatively supercoiled DNA topoisomers. A schematic drawing of a two-dimensional gel is shown in Figure 5A. Both GyrA-3xA mutant enzymes lost the ability to supercoil DNA in the presence of ATP (Figure 5B).

**Figure 5:**
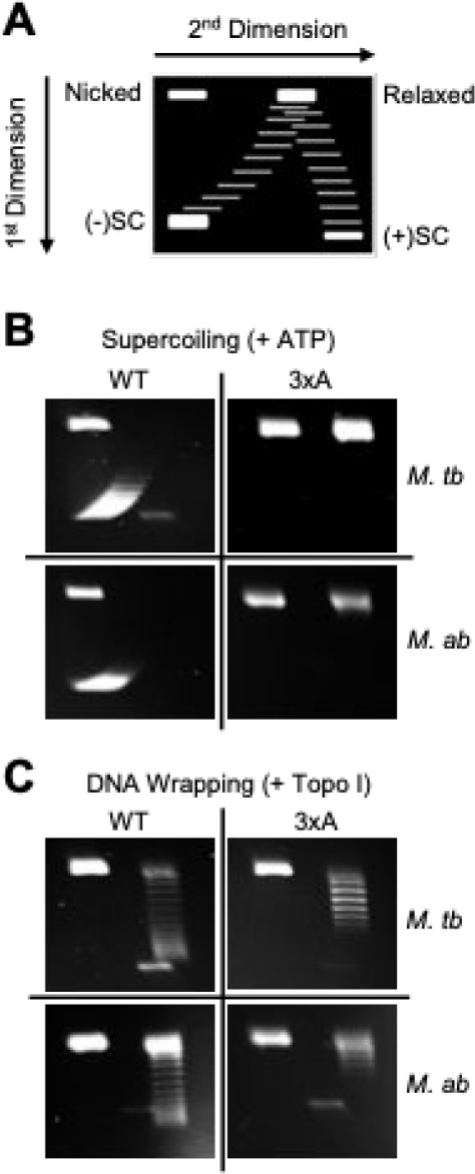
Effects of three glycine (G) to alanine (A) substitutions in the GyrA-box (GyrA-3xA mutant) of *M. tuberculosis* and *M. abscessus* gyrase. (A) A schematic drawing of a two-dimensional gel separating positively supercoiled [(+)­SC] from negatively supercoiled topoisomers [(−)­SC]. (B) Products of supercoiling reactions with wild-type (WT, left) and GyrA-3xA mutant (3xA, right) gyrase from *M. tuberculosis* (*M. tb*, top) and *M. abscessus* (*M. ab*, bottom), separated by two-dimensional gel electrophoresis. (C) To assess whether substituting three glycine residues in the GyrA-box with alanine residues altered the ability of the enzyme to wrap DNA, wild-type (WT) or mutant (3xA) gyrase heterotetramer (800 nM *M. tuberculosis*, top; 400 nM *M. abscessus*, bottom) was incubated with relaxed pBR322 in the absence of ATP and treated with topoisomerase I to relax compensatory negative supercoils formed by gyrase wrapping of positive DNA writhes. Products are shown on two-dimensional gels.

To assess whether the GyrA-3xA mutant enzymes lost the ability to wrap DNA, wild-type or mutant gyrase was incubated with relaxed plasmid in the absence of ATP and subsequently treated with topoisomerase I to relax compensatory negative supercoils formed by gyrase wrapping of positive DNA writhes. Compared to wild-type, the GyrA-3xA mutant enzymes displayed significantly reduced DNA wrapping (Figure 5C).

A caveat to this conclusion is the fact that wild-type gyrase reportedly has a weak ability to relax negative (but not positive) supercoils in the absence of ATP.^24,63,64^ We found that this was the case for both mycobacterial gyrases tested, but that the GyrA-3xA mutant enzymes lost this ATP-independent relaxation activity (data not shown). Consequently, the ability of the wild-type but not the mutant gyrases to remove compensatory negative supercoils could potentially skew results making it appear that the wild-type enzymes wrap DNA more than the enzymes with substitutions in the GyrA-box. However, the amount of topoisomerase I added to remove compensatory negative supercoils displayed a relaxation activity that was at least 2 orders of magnitude greater than that of the wild-type gyrases (data not shown). Thus, the weak ATP-independent DNA relaxation activity of wild-type gyrases vs the mutant enzymes should not affect the conclusions of the DNA wrapping experiments shown in Figure 5C.

Having determined that the DNA-wrapping ability of the GyrA-3xA mutant enzymes was substantially reduced, we compared the abilities of the wild-type and mutant enzymes to decatenate kDNA. As predicted, the GyrA-3xA mutation in *M. tuberculosis* gyrase resulted in an increased rate of decatenation (Figure 6, left panel). In contrast, the GyrA-3xA mutation in *M. abscessus* gyrase did not affect the rate of decatenation (Figure 6, right panel), despite the fact that it could no longer wrap DNA. This finding was unexpected given the high degree of sequence similarity between *M. tuberculosis* and *M. abscessus* gyrase. However, it afforded a unique opportunity to determine if rate was the primary factor influencing levels of drug required to inhibit decatenation versus intramolecular DNA supercoiling or relaxation.

**Figure 6:**
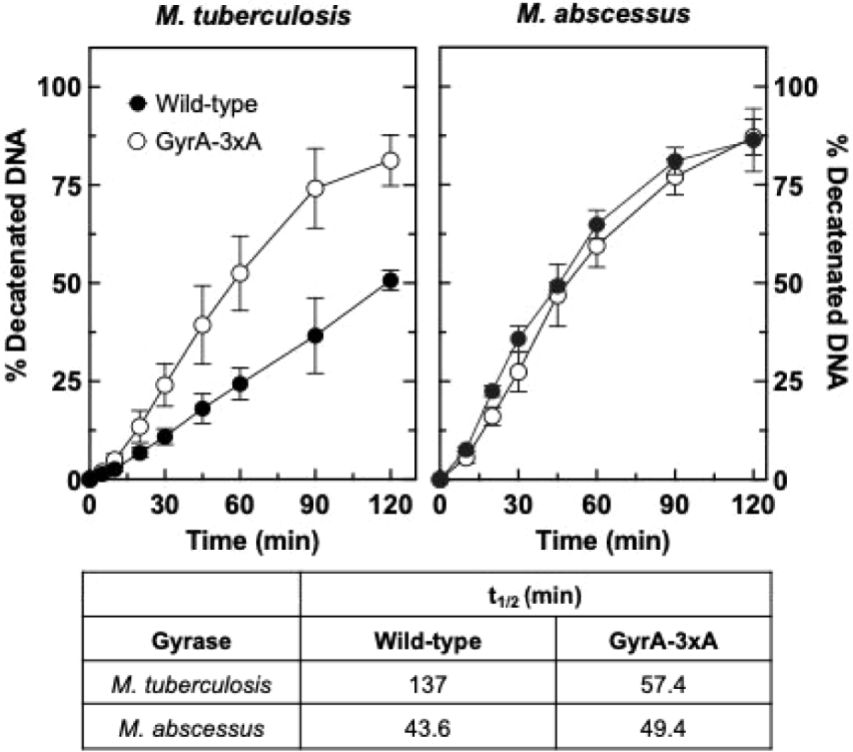
Effects of GyrA 3xA-box mutation on rates of decatenation catalyzed by *M. tuberculosis* and *M. abscessus* gyrase. Results of decatenation time courses with wild-type (closed circles) and GyrA-3xA mutant (open circles) gyrase from *M. tuberculosis* (left panel) and *M. abscessus* (right panel) are shown. Error bars represent the standard deviation of at least three independent experiments. Half-times (*t*_1/2_) are listed in minutes in the table at the bottom of the figure.

As seen in Figure 7, higher concentrations of moxifloxacin, ciprofloxacin, and zoliflodacin were required to inhibit DNA decatenation catalyzed by the mutant *M. tuberculosis* enzyme compared to wild-type gyrase. These findings suggest one of two possibilities: an increase in reaction rate leads to higher inhibitory concentrations for the decatenation of kDNA, or the lack of wrapping may affect the geometry of DNA in the active site, thereby changing the interaction of the antibacterials with the enzyme-DNA complex. To determine which explanation best reflects the basis for reduced inhibition, we determined the effects of the three antibacterials on decatenation catalyzed by the *M. abscessus* GyrA-3xA mutant enzyme (Figure 8), which decatenates at the same rate as wild-type (see Figure 6, right panel). Results were similar to those seen with the *M. tuberculosis* enzymes. Higher concentrations of all three antibacterials were required to inhibit decatenation catalyzed by the *M. abscessus* GyrA-3xA mutant compared to the wild-type enzyme.

**Figure 7:**
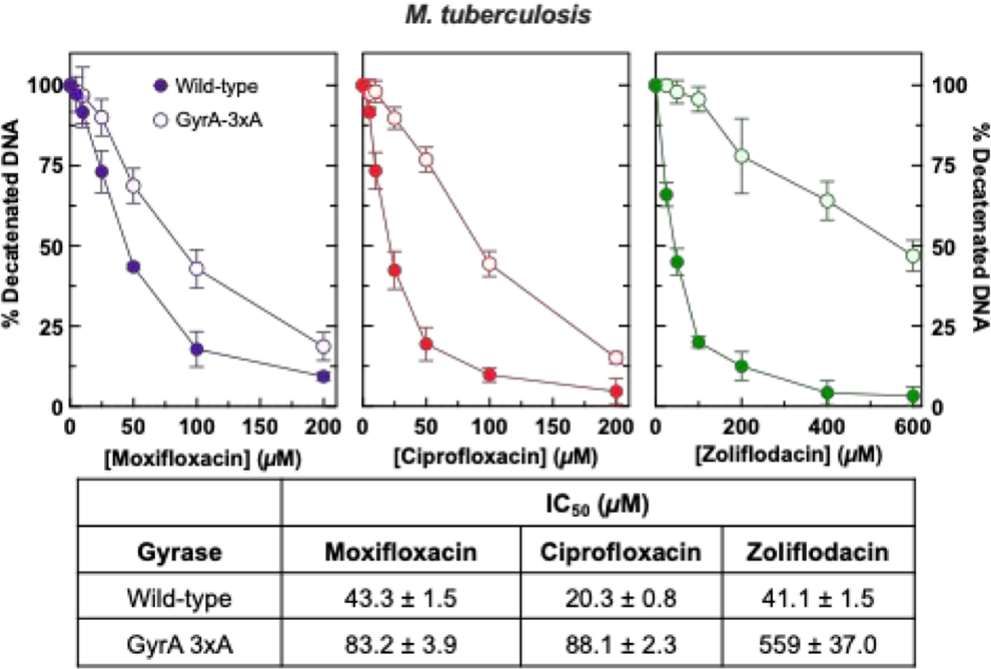
Inhibition of decatenation catalyzed by wild-type and GyrA-3xA mutant gyrase from *M. tuberculosis*. The ability of wild-type gyrase (closed circles) and GyrA-3xA mutant gyrase (open circles) from *M. tuberculosis* to catalyze decatenation in the presence of moxifloxacin (purple, left panel), ciprofloxacin (red, middle panel), and zoliflodacin (green, right panel) is shown. Error bars represent the standard deviation of at least three independent experiments. IC_50_ values are listed in μM with the standard error of the mean in the table at the bottom of the figure.

**Figure 8:**
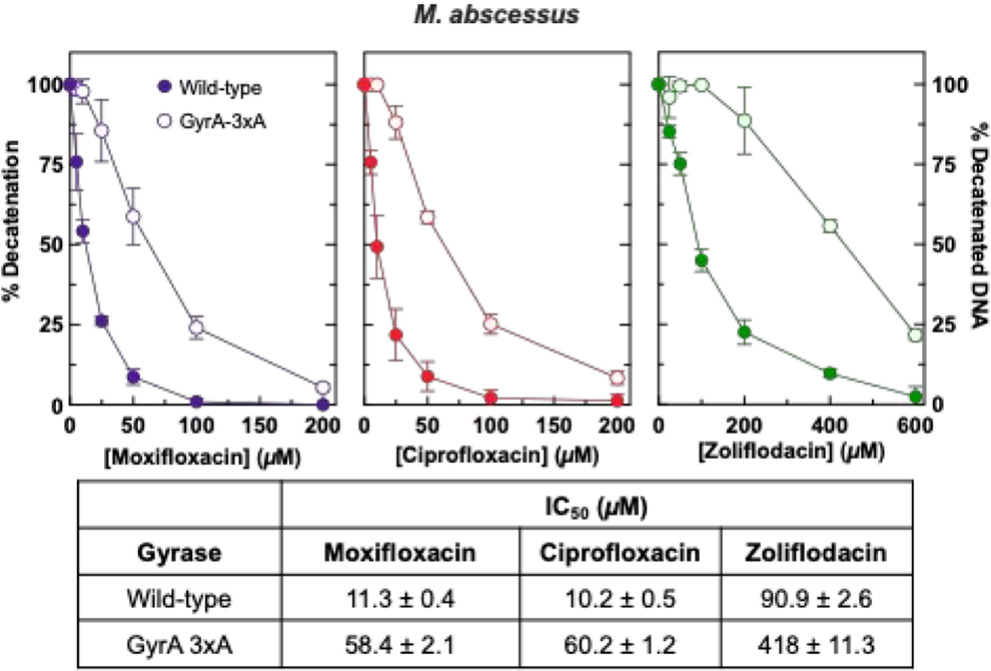
Inhibition of decatenation catalyzed by wild-type and GyrA-3xA mutant gyrase from *M. abscessus*. The ability of wild-type gyrase (closed circles) and GyrA-3xA mutant gyrase (open circles) from *M. abscessus* to catalyze decatenation in the presence of moxifloxacin (purple, left panel), ciprofloxacin (red, middle panel), and zoliflodacin (green, right panel) is shown. Error bars represent the standard deviation of at least three independent experiments. IC_50_ values are listed in μM with the standard error of the mean in the table at the bottom of the figure.

This finding precludes the explanation that differences in rates between different reactions are the primary driver of drug potency. Instead, it suggests that the ability of interfacial antibacterials^36^ to inhibit gyrase-catalyzed reactions is dependent on the specific interactions between the DNA and the enzyme. In the case of the GyrA-3xA mutant, it is unknown whether it is the lack of wrapping *per se* or a subtle difference in the structure of the enzyme active site caused by the loss of GyrA-box function that leads to the observed differences in inhibition.

### Effects of DNA Substrate on Inhibition of Gyrase by Novobiocin

If the potency of interfacial antibacterials is dependent upon enzyme–DNA interactions, we would predict that an antibacterial that does not simultaneously interact with the enzyme and DNA would not be affected by different substrates and rates of reactions catalyzed by gyrase. To test this prediction, we examined the effects of novobiocin on the relaxation of positive supercoils, the introduction of negative supercoils into relaxed DNA, and the decatenation of kDNA catalyzed by *M. tuberculosis* and *M. abscessus* gyrase. In contrast to the interfacial inhibitors (moxifloxacin, ciprofloxacin, and zoliflodacin), novobiocin (an aminocoumarin) is an inhibitor of the ATPase activity of gyrase and binds to the B subunit, distal from the DNA cleavage-ligation active site.^65,66^ Novobiocin has no known interaction with DNA in the gyrase-DNA complex.

As seen in Figure 9, only minimal differences were observed in IC_50_ values for the inhibition of the three catalytic functions of gyrase by novobiocin. Furthermore, near-identical IC_50_ values were calculated when comparing the inhibition of decatenation catalyzed by wild-type vs GyrA-3xA mutant enzymes from *M. tuberculosis* and *M. abscessus* (Figure 10). These findings support the hypothesis that the inhibition of gyrase-catalyzed reactions by antibacterials that interact with both the enzyme and DNA is related to the nature of the DNA substrate and its interactions with gyrase. Additionally, the IC_50_ values calculated for the comparable enzymes from both bacteria were remarkably similar in all reactions. This consistency likely reflects the conservation of the ATP binding sites in both species and the fact that, unlike interfacial poisons, novobiocin–enzyme interactions are independent of enzyme–DNA interactions.

**Figure 9:**
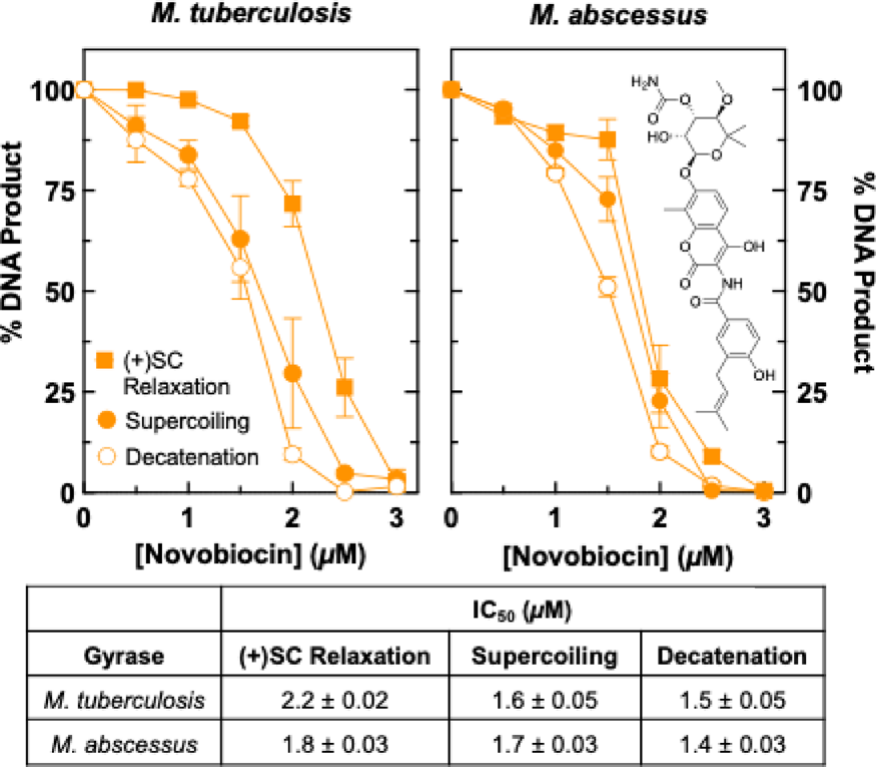
Inhibition of the three catalytic activities of *M. tuberculosis* and *M. abscessus* gyrase by novobiocin. The ability of *M. tuberculosis* (left panel) and *M. abscessus* (right panel) gyrase to catalyze decatenation of kDNA (Decatenation, open circles), negative supercoiling of relaxed DNA (Supercoiling, closed circles), and relaxation of positive supercoils [(+)­SC Relaxation, closed squares] in the presence of increasing concentrations of novobiocin is shown. The structure of novobiocin is shown in the right panel. Error bars represent the standard deviations of at least three independent experiments. IC_50_ values are listed in μM with the standard error of the mean in the table at the bottom of the figure.

**Figure 10:**
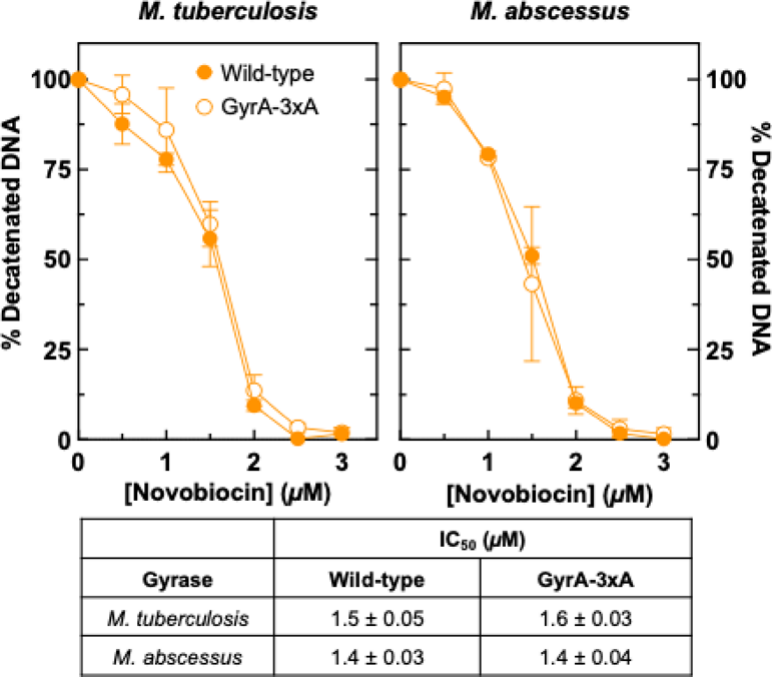
Inhibition of decatenation catalyzed by wild-type and GyrA-3xA mutant gyrases from *M. tuberculosis* and *M. abscessus* by novobiocin. The ability of wild-type gyrase (closed circles) and GyrA-3xA mutant gyrase (open circles) from *M. tuberculosis* and *M. abscessus* to catalyze decatenation in the presence of novobiocin is shown in the left and right panels, respectively. Error bars represent the standard deviation of at least three independent experiments. IC_50_ values are listed in μM with the standard error of the mean in the table at the bottom of the figure.

## Discussion

Gyrase and topoisomerase IV play critical roles during DNA replication.^5–7^ Gyrase removes positive supercoils that accumulate ahead of replication forks, while topoisomerase IV untangles daughter chromosomes, which allows chromosome segregation to take place during cell division. Topoisomerase IV is an essential enzyme in bacteria that encode it.^14^ However, some bacteria, such as *M. tuberculosis* and *M. abscessus*, encode only gyrase, which presumably plays the role of both enzymes in these species.^26–29^ In *M. tuberculosis* and *M. abscessus*, gyrase is the sole target for fluoroquinolones and spiropyrimidinetriones.^33,34,37,52,67,68^ Although these antibacterials induce gyrase-mediated DNA cleavage,^34,35,52,68^ there is evidence that inhibition of gyrase function also plays a role (at least under some circumstances) in drug-induced cell death.^37^ Therefore, we examined the effects of moxifloxacin, ciprofloxacin, and zoliflodacin on the three catalytic activities of gyrase; decatenation, negative supercoiling of relaxed DNA, and relaxation of positive supercoils. Under all circumstances, lower concentrations of antibacterials were required to inhibit intermolecular DNA decatenation as compared to the intramolecular DNA relaxation or supercoiling functions of gyrase. This finding suggests that the major phenotype of fluoroquinolone (and other antibacterials) action on the cellular function of gyrase in actively replicating *Mycobacteria* would likely be impaired chromosome partitioning as opposed to decreased rates of replication. Indeed, treatment of *Mycobacterium smegmatis* with moxifloxacin^69^ leads to a cellular morphology that resembles the partitioning defects that result from a loss of topoisomerase IV in bacteria that encode this second type II topoisomerase.^14^

Results suggest that the potency of gyrase inhibition by interfacial antibacterials is modulated by the topological state of the DNA and its specific interactions with gyrase rather than the rate at which different reactions are catalyzed. We consistently observed that intermolecular DNA passage reactions (i.e., decatenation) were inhibited at lower concentrations of fluoroquinolones and spiropyrimidinetriones than intramolecular DNA passage reactions (i.e., supercoiling and relaxation). It is unclear why different DNA substrates would have this effect on drug activity. One possibility is that intramolecular DNA substrates are either under torsional stress or are incurring torsional stress during strand passage. During both reactions (supercoiling and relaxation), DNA wrapping plays a major role. In contrast, the decatenation of kDNA never involves the generation of torsional stress, as kDNA is an intrinsically relaxed substrate.^70^ Additionally, DNA wrapping should hinder decatenation as it causes gyrase to favor intramolecular strand passage. Clearly, both the torsional stress of supercoiling and the axial stress of DNA wrapping have the potential to alter drug interactions in the active site of the enzyme-DNA complex. Future structural studies of enzyme-drug--DNA complexes will be required to resolve this issue. At the present time, there is only one gyrase structure that includes a DNA substrate that is under torsional stress (negatively supercoiled DNA) and there is no drug in the complex.^71^

Ultimately, most studies focus on DNA cleavage induced by gyrase-targeted antibacterials. In contrast, the present study provides mechanistic insights into how these drugs rob replicating cells of the essential functions of this critical enzyme in species that do not encode a separate decatenase.

## Materials and Methods

### DNA Substrates

Negatively supercoiled pBR322 was expressed in *E. coli* and purified using a Qiagen Mega Kit according to the manufacturer’s protocol. The relaxed pBR322 used in supercoiling and DNA wrapping experiments was generated by incubating 50 μg of negatively supercoiled pBR322 with 32 units of calf thymus topoisomerase I (Invitrogen) in a total volume of 150 μL of topoisomerase I buffer [10 mM Tris-HCl (pH 7.9), 0.1 mM Na_2_EDTA, 175 mM KCl, 5 mM MgCl_2_, and 2.5% glycerol] at 37 °C for 1 h followed by a 15 min heat inactivation at 75 °C. The preparation of positively supercoiled pBR322 was based on previously published protocols.^72^ Briefly, 50 μg of negatively supercoiled pBR322 was incubated with 35 μg of reverse gyrase (purified according to the protocol of Rodriguez^73^) in 500 μL of a buffer solution containing 50 mM Tris-HCl (pH 8.0), 10 mM NaCl, 10 mM MgCl_2_, and 0.3 mM ATP at 95 °C for 5 min. The reaction was treated with proteinase K to digest reverse gyrase, DNA was extracted using phenol:chloroform:isoamyl alcohol (25:24:1), precipitated with ice-cold 100% ethanol, resuspended in 5 mM Tris-HCl (pH 7.4) and 500 μM EDTA, and purified using Micro Bio-Spin Columns (BioRad) according to the manufacturer’s protocol. kDNA was purified from *C. fasciculata* according to the protocol of Englund.^60^ All DNA substrates were kept at −80 °C for long-term storage, then at 4 °C for short-term storage after thawing.

### Enzymes

6x His-tagged *M. tuberculosis* wild-type gyrase subunits (GyrA and GyrB) were purified by nickel affinity chromatography as described by Aldred et al.,^52^ with the exclusion of the TEV protease cleavage step. *M. abscessus* GyrA and GyrB were custom-ordered from GenScript Biotech Corporation. Briefly, wild-type *M. abscessus* GyrA and GyrB sequences were separately cloned into pET31a vectors with attached N-terminal 6x-Histidine/TEV protease sequences, which were then expressed and purified from *E. coli* by GenScript. Mutant GyrA subunits were cloned using a QuikChange site-directed mutagenesis kit (Agilent) and purified according to the protocol of Aldred et al.^52^ with the exclusion of the TEV protease cleavage step. All enzyme subunits were stored at −80 °C. Immediately prior to use, gyrase subunits were thawed and diluted to desired concentrations in a buffer containing 50 mM HEPES-KOH (pH 7.6), 300 mM KCl, and 30% glycerol.

### Antibacterials

All compounds were stored in solution at −20 °C. Moxifloxacin-HCl (Sigma) and zoliflodacin (MedChemExpress) were stored as 60 mM stock solutions in 100% DMSO. Ciprofloxacin (Sigma) was stored as a 40 mM stock solution in 0.1 N NaOH. Novobiocin (Sigma) was stored as a 20 mM stock solution in ultrapure H_2_O. Moxifloxacin was diluted 10-fold with water to generate a 6 mM solution in 10% DMSO and serially diluted to 10x the desired final concentration with 10% DMSO. Ciprofloxacin was diluted 5-fold with 10 mM Tris-HCl (pH 7.9) to generate an 8 mM working stock in 0.02 M NaOH and 8 mM Tris-HCl (pH 7.9). Serial dilutions were performed using a solution of 0.02 M NaOH and 8 mM Tris-HCl (pH 7.9). Zoliflodacin was diluted with water and DMSO to a concentration of 6 mM in 40% DMSO and subsequently diluted in 40% DMSO. Novobiocin was diluted in ultrapure water.

### Time Course Experiments

All reactions were carried out using 0.3 μg of DNA (kDNA for decatenation, relaxed pBR322 for supercoiling, and positively supercoiled pBR322 for relaxation) in a 20 μL total volume of gyrase buffer [10 mM Tris-HCl (pH 7.5), 40 mM KCl, 6 mM MgCl_2_, 0.1 mg/mL bovine serum albumin, 10% glycerol, 5 mM dithiothreitol] plus 1 mM ATP. Gyrase subunits were diluted to 20x the desired final concentration of holoenzyme in a buffer containing 50 mM HEPES-KOH (pH 7.6), 300 mM KCl, and 30% glycerol. The final concentration of gyrase holoenzyme was 200 nM (2:1 GyrA:GyrB ratio) for *M. tuberculosis* gyrase and 50 nM (1:1 GyrA:GyrB ratio) for *M. abscessus* gyrase. Reactions were incubated at 37 °C for varying amounts of time and stopped by the addition of 2 μL of 250 mM Na_2_EDTA (pH 8.0). Samples were incubated at 85 °C for 10 min to allow gyrase to religate cleaved DNA intermediates. Two μL of 5% sodium dodecyl sulfate (SDS) and 2 μL of agarose gel loading buffer (600 mg/mL sucrose, 10 mM Tris-HCl (pH 7.9), 5 mg/mL bromophenol blue, 5 mg/mL xylene cyanol) were added. Samples were subjected to electrophoresis at approximately 7.5 V/cm in a 1% agarose gel in Tris-Borate-EDTA buffer. Gels were stained with ethidium bromide, and DNA was visualized with midrange ultraviolet light using an AlphaImager (Protein Simple). DNA bands were quantified using AlphaView (Alpha Innotech). (+)­SC relaxation was quantified as the loss of the substrate present at time zero. Supercoiling was quantified as a percentage of the fully supercoiled DNA at the final time point. Decatenation was quantified as a percentage based on a standard containing 0.3 μg of fully decatenated kDNA. Half-times (*t*_1/2_ values) were calculated using a one-phase association curve fit in GraphPad Prism.

### Catalytic Inhibition of Gyrase

Catalytic inhibition experiments were carried out under the same conditions as time courses. Drugs were diluted to 10x the desired final concentration using the appropriate diluent, and 2 μL of 10x drug was added to 18 μL of gyrase buffer containing the gyrase holoenzyme [200 nM (2:1 GyrA:GyrB ratio) for *M. tuberculosis* gyrase and 50 nM (1:1 GyrA:GyrB ratio) for *M. abscessus* gyrase], 0.3 μg of DNA (kDNA for decatenation, relaxed pBR322 for supercoiling, and positively supercoiled pBR322 for relaxation), and 1 mM ATP. Two control reactions were included in each experiment: one containing DNA with no enzyme and one containing DNA and enzyme with 2 μL of the same diluent as the 10x drug. All reactions (including controls) contained an equivalent concentration of the same diluent as the specific drug being tested to allow for direct comparisons. Samples were incubated at 37 °C for the following amounts of time, depending upon enzyme and reaction: decatenation reactions were incubated for 90 min, supercoiling reactions for 15 min (*M. tuberculosis* gyrase) or 10 min (*M. abscessus* gyrase), and relaxation reactions for 2 min (*M. tuberculosis* gyrase) or 1.75 min (*M. abscessus* gyrase). In the presence of ciprofloxacin diluent, wild-type *M. tuberculosis* gyrase relaxed positively supercoiled DNA slightly faster than in the presence of DMSO. Therefore, we adjusted the positively supercoiled DNA relaxation reaction time for ciprofloxacin titrations to 1.5 min with *M. tuberculosis* gyrase. Reactions were stopped and processed as described above. IC_50_ values were calculated in GraphPad Prism using an [Inhibitor] vs normalized response curve fit.

### DNA Wrapping

To monitor DNA wrapping by the C-terminal domain of gyrase, 0.3 μg of relaxed pBR322 was incubated with high concentrations of the wild-type or mutant gyrase from *M. tuberculosis* or *M. abscessus* (800 nM for *M. tuberculosis*, 400 nM for *M. abscessus*) in the absence of ATP. Enzyme subunits and DNA were combined in a final volume of 20 μL of gyrase buffer and incubated at 37 °C for 15 min. Following the initial incubation, 2 μL of topoisomerase I diluted to 0.08 units/μL in topoisomerase I buffer was added to remove compensatory negative supercoils induced by the formation of gyrase-stabilized positive supercoils. Reactions were incubated at 37 °C for an additional 25 min. Reactions were stopped and processed as described above and resolved by two-dimensional gel electrophoresis.

### Two-Dimensional Gel Electrophoresis

Two-dimensional gel electrophoresis was carried out according to the protocol of Gibson et al.^74^
